# Double aortic arches, esophageal atresia and tracheal compression

**DOI:** 10.4103/0971-9261.55157

**Published:** 2009

**Authors:** Yameen Majid, Monali Warade, Zarina Aziz, G. A. Karthik

**Affiliations:** Department of Radiodiagnosis, Narayana Hrudayalaya, Institute of Medical Sciences, Bangalore, India

**Keywords:** Double aortic arch, congenital heart defects, esophageal atresia, vascular rings, computed tomography, helical

## Abstract

We report a case of double aortic arch in a 12-month-old male infant well delineated on 64 slice computed tomography scan. It formed a complete vascular ring around the trachea compressing it. The symptoms resolved after surgical division of the ring.

## INTRODUCTION

Double aortic arch refers to the congenital anomaly in which both the aortic arches remain patent and form a complete vascular ring encircling the trachea and esophagus.[1] It is a very rare cause of complete vascular ring in which both the right and left aortic arches are present to various extents and usually result in noncardiac morbidity especially compression of trachea.[2] Not many cases are reported in literature of this anomaly. Further, a complete tracheal ring with both functional arches is the rarest of all types of double aortic arch. We report a case of complete double aortic arch with classical manifestations and very well delineated on 64 slice computed tomography (CT) with complete resolution of symptoms after surgical correction.

## CASE REPORT

A 12-month-old male infant presented with history of stridor. He was born at 39 weeks gestation and weighed 2.5 kg. APGAR score was normal at one and five minutes. The baby was previously operated for esophageal atresia and tracheo esophageal fistula (EA-TEF) (type 1 - communication between the lower pouch of esophagus with trachea) at the age of three days at another hospital but the stridor persisted and worsened with time. In addition the patient developed recurrent episodes of dysphagia, cyanotic episodes and respiratory tract infection. At six months the patient was admitted again because of severe stridor and dyspnoea. Clinical examination on patient showed mild respiratory distress, respiratory rate of 55/min, pulse rate of 122/min, SPO2 of 93% with oxygen by hood and a blood pressure of 75/50. On systemic examination the patient showed normal air conduction on both sides with mild bilateral crackles.

**Figure 1:**
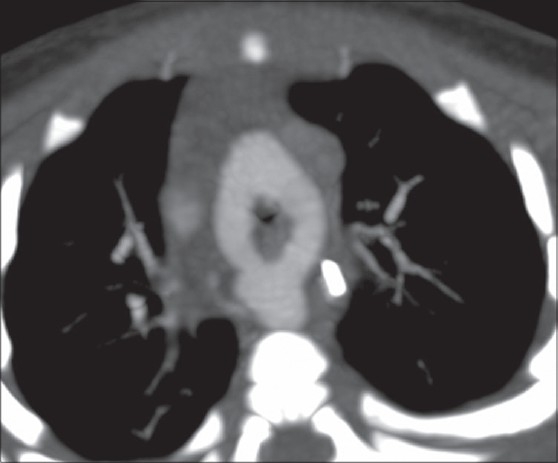
Axial CT chest shows double aortic arch

**Figure 2:**
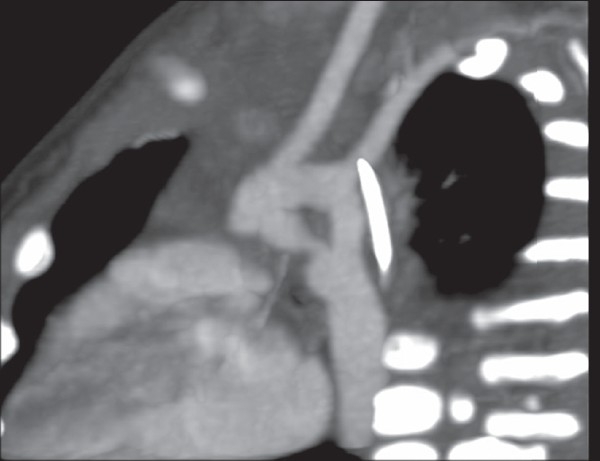
Oblique reformats two arches unite to form single descending aorta

**Figure 3:**
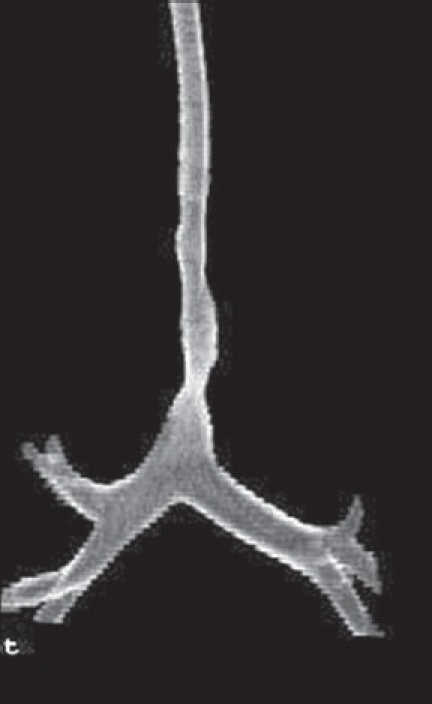
Virtual bronchoscopy demonstrates tracheal compression

**Figure 4:**
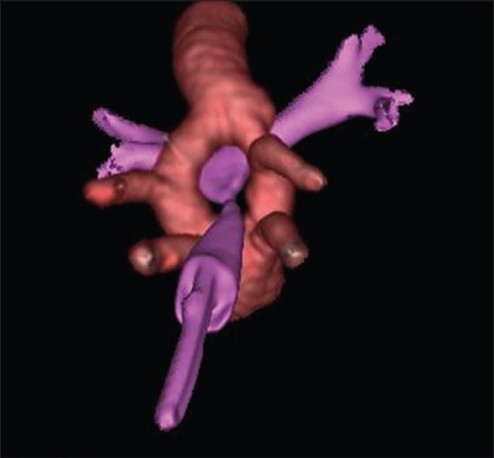
3D reconstruction: Double aortic arch encircling trachea Red – Aorta, Pink- Trachea

Bronchoscopy revealed compression of the trachea along with laryngomalacia. Echocardiography did not reveal any significant abnormality. A suspicion of vascular ring was made on clinical grounds and bronchoscopy findings and a contrast CT of chest was advised. Contrast enhanced helical CT was done using 64-slice Light speed VCT (GE Medical systems, Milwaukee, Wisconsin). Maximum intensity projection (MIP) and volume rendered (VR) images were also obtained for better delineation of the anatomy. A complete functional double aortic arch forming a vascular ring around the caudal aspect of trachea at the level of D4 vertebra was seen causing significant narrowing of the trachea at that level for a short segment of 0.6cm at about 5mm proximal to the bifurcation. [Figure 1–4]. The right sub clavian and common carotid arteries showed separate origins from the right aortic arch and left sub clavian and common carotid arteries showed separate origins from the left aortic arch. [Figures 2 and 4] The two arches were seen to unite approximately five mm above the level of carina to form a single descending aorta. [Figure 2] After five days, the patient was operated through a posterolateral thoracotomy and the presence of double aortic arch was confirmed. Ligation of the left arch was done, distal to the subclavian artery. He was discharged home one week after this procedure. He had persistent mild stridor in the postoperative period. There was no stridor at six months follow-up.

## DISCUSSION

The first reference of double aortic arch goes back to 1737 by Hommel. Not much literature is available on double aortic arch; however there have been few case reports of double aortic arch in association with other anomalies. One such case was reported by Talwar *et al*.[3] in 2009. In their case a complete double aortic arch was associated with Tetralogy of Fallot and absent left pulmonary artery. Another case of double aortic arch has been reported by Lotz[4] in a six-month-old boy who had recurrent respiratory difficulty. In our case double aortic arch was associated with esophageal atresia and tracheo esophageal fistula.

The usual presentation of double aortic arch is due to tracheal compression leading to symptoms of upper airway obstruction in the form of stridor or dyspnoea.[5] In infants the early symptoms may also be harsh breathing noted by parents. The patients may also present with symptoms similar to lower respiratory infections, so respiratory viral studies are usually done to exclude infectious etiology. However, as double arch predisposes patients to viral infections the diagnosis of an infection does not exclude the possibility of double aortic arch. A routine chest radiograph may reveal indentation of the tracheal air column. Echocardiography is not usually done initially because of the presentation of the patient is with respiratory tract infections, and usually the diagnosis is made on cross sectional imaging like Magnetic Resonance Imaging (MRI)[6] and CT or barium esophagogram. However, echocardiography can reliably diagnose double aortic arch and also is important in evaluation of associated cardiovascular anomalies. Barium esophagogram usually shows bilateral indentation of the esophagus. MRI and CT are best imaging modalities for diagnosis and characterization of double aortic arch and provide in addition complete information on arterial branching pattern, extent of tracheal and esophageal obstruction and preoperative planning. Bronchoscopy may be done in some patients in the evaluation of airway pathologic condition which shows pulsatile compression of the posterior and lateral walls of the trachea

Double aortic arches are classified into two types depending upon the patency of the two arches: type 1, which has both arches functioning, type 2 which has one of the arches atretic and is further classified into four subtypes depending upon location of the atretic segment.[7] In our case a completely functioning double aortic arch was seen. Treatment is usually surgical, the most common being surgical ligation of the small arch distal to sub clavian artery.
